# Impact of individual socioeconomic deprivation on hemodialysis care and patient behavior: a multicenter French study (Precadia)

**DOI:** 10.1093/ckj/sfaf201

**Published:** 2025-06-26

**Authors:** Yves Dimitrov, François Chantrel, Thierry Krummel, Clotilde Muller, Emmanuelle Charlin, Alexandre Klein, Thierry Hannedouche

**Affiliations:** Department of Nephrology, CHH, Haguenau, France; Department of Nephrology, GHMSA, Mulhouse, France; Department of Nephrology, HUS, Strasbourg, France; Department of Nephrology, GHSV, Strasbourg, France; Dialysis Center, AURAL, Strasbourg, France; Department of Nephrology, HCC, Colmar, France; Dialysis Center, AURAL, Strasbourg, France

**Keywords:** EPICES score, health inequities, hemodialysis, patient behavior, socioeconomic deprivation

## Abstract

**Background:**

Socioeconomic deprivation (SED) is increasingly recognized as a key determinant of morbidity and mortality among patients receiving hemodialysis. However, most prior studies have relied on area-level socioeconomic indicators and have been conducted outside of Europe.

**Objective:**

To assess whether individual-level socioeconomic status, measured using the EPICES score, influences healthcare quality and patient behaviors in adults undergoing maintenance hemodialysis in northeastern France.

**Methods:**

This multicenter observational study was conducted across five dialysis units. Adult patients with end-stage renal disease receiving hemodialysis for ≥3 months were enrolled. The EPICES score was used to assess individual SED, and patients were stratified into deprived (P+) and non-deprived (P−) groups based on the median score. Clinical, biochemical, and behavioral variables were compared between groups.

**Results:**

A total of 401 patients were included (mean age 68.5 years; 60% male). The median EPICES score was 33.1 (mean 35.8 ± 18.9). Compared with P − patients, P + patients had a significantly longer dialysis vintage (76.3 vs. 73.9 months, *p* = .002), higher normalized protein catabolic rate (nPCR; 1.28 vs. 1.06 g/kg/day, *p* = .007), higher CRP concentrations (14.3 ± 4.1 vs. 9.56 ± 0.8 mg/L, *p* < .02 ), and greater smoking prevalence (34% vs. 15%, *p* = .004). No significant differences were observed in albumin and hemoglobin levels, erythropoietin (EPO) dosing, or the frequency of missed dialysis sessions. The CRP × P + interaction on nPCR was significant, suggesting that systemic inflammation could modify the association between deprivation and protein catabolism.

**Conclusion:**

Individual-level SED was associated with differences in health behaviors but not in the quality of physician-driven dialysis care. These findings challenge the notion that deprived patients inherently receive lower-quality clinical care and emphasize the need for targeted strategies that address patient-dependent behavioral factors.

KEY LEARNING POINTS **What was known**:

- Socioeconomic deprivation (SED) has been linked to poorer outcomes in hemodialysis patients, but most evidence relies on area-level indicators and originates from non-European healthcare systems.
- The influence of individual-level SED on both healthcare delivery and patient behavior in settings with universal healthcare coverage, like France, remained underexplored.
- Prior studies did not distinguish whether worse outcomes stemmed from inequalities in medical care or from patient-related behavioral factors.

**This study adds**:

- Using the EPICES score, this study found that individual SED is not associated with disparities in physician-delivered dialysis care such as anemia management or dialysis adequacy.
- Socioeconomically deprived patients displayed higher inflammation (CRP), greater smoking rates, and elevated normalized protein catabolic rates, suggesting behavioral and biological differences.
- These findings emphasize that disparities in outcomes may be driven more by patient-level behaviors than by inequity in healthcare provision.

**Potential impact**:

- The study supports the equity of dialysis care delivery within the French healthcare system, even across socioeconomic strata.
- It highlights the importance of targeting behavioral and inflammatory risk factors—like smoking and chronic inflammation—among deprived patients to improve outcomes.
- Incorporating individualized social risk assessments like the EPICES score can guide tailored interventions aimed at modifiable behavioral contributors to poor prognosis.

## INTRODUCTION

Patients with chronic kidney disease undergoing hemodialysis are subject to a high burden of complications and face elevated mortality rates [1]. While numerous clinical and biological risk factors for morbidity and mortality have been well established in this population, socioeconomic deprivation (SED) has more recently emerged as a relevant determinant of outcomes [2–6], as reviewed in detail by Couchoud et al. [7]. Most of these studies suggest that various dimensions of deprivation—including low income, unemployment, and limited educational attainment—are associated with higher mortality among hemodialysis patients.

However, the generalizability of these findings is limited. Nearly all such studies, with the notable exception of one conducted in France [4], were carried out internationally, predominantly outside of Europe. Comparing findings across countries is challenging due to significant differences in healthcare organization and the extent of social protection, which may influence access to care and treatment outcomes. Interestingly, the only study that did not observe an association between SED and mortality in dialysis patients was conducted within the French healthcare system [4], where universal health coverage may attenuate the impact of socioeconomic disparities.

Another critical limitation of the existing literature is the method used to assess socioeconomic status (SES). In most studies, SES was inferred from area-level indicators—typically the average socioeconomic profile of the patient's residential neighborhood—rather than from individual-level data. Additionally, these composite indices have often been based primarily on economic parameters such as neighborhood income levels or unemployment rates, which may not fully capture the multifaceted nature of individual deprivation.

The mechanisms by which SED might influence morbidity and mortality in dialysis patients remain under investigation. One hypothesis is that deprivation compromises treatment

adherence, leading to missed dialysis sessions, poor medication compliance, and failure to follow dietary restrictions—all of which are associated with worse clinical outcomes [8, 9]. Alternatively, it has been suggested that patients experiencing deprivation may receive suboptimal care from healthcare providers, particularly with regard to timely access to transplantation and other advanced therapies [10].

The present study seeks to address these gaps by evaluating the relationship between individual-level socioeconomic deprivation and the quality of care received by hemodialysis patients. Using a validated questionnaire-based assessment of SES, we aimed to distinguish between care indicators dependent on the healthcare team and those influenced by patient behavior. Our goal was to determine whether individual socioeconomic status affects the delivery and/or adherence to dialysis care in a setting of universal healthcare coverage.

## MATERIALS AND METHODS

This observational and multicenter study took place in five hemodialysis centers located in the same region of northeastern France. The inclusion criterion was as follows: adult patients with end-stage renal disease treated with hemodialysis for at least 3 months, on a thrice weekly schedule. The exclusion criteria were: minor patients, inability or refusal to complete the questionnaire, and those residing in institutions (as the questionnaire was not validated for this population).

Socioeconomic deprivation was assessed using the EPICES score (Table 1). The score consists of 11 binary questions (yes/no) that analyze economic as well as social deprivation, particularly isolation. Each question is weighted by a positive or negative coefficient, and their sum defines a deprivation score. This score ranges from 0 (no deprivation) to 100 (extreme deprivation). The threshold for deprivation was established at 30 in the French population [11]. The EPICES questionnaire was developed in a French population of 197 389 healthy individuals [12]. It was subsequently validated in various populations with different pathologies and used in epidemiological studies of chronic diseases such as diabetes [13, 14], obesity [15], stroke [16], peripheral artery disease, and coronary artery disease [17].

**Table 1:** EPICES score: components and calculation.

N°	Questions	Yes	No
1	Do you sometimes meet with a social worker?	10.06	0
2	Do you benefit from supplementary health insurance?	−11.83	0
3	Do you live with a partner?	−8.28	0
4	Are you a homeowner?	−8.28	0
5	Are there times during the month when you face serious financial difficulties in meeting your basic needs (food, rent, electricity, etc.)?	14.80	0
6	Have you engaged in any sporting activity during the past 12 months?	−6.51	0
7	Have you attended a cultural event (e.g. theater, concert) during the past 12 months?	−7.10	0
8	Have you gone on vacation during the past 12 months?	−7.10	0
9	In the past 6 months, have you had contact with family members other than your parents or children?	−9.47	0
10	In case of difficulties, do you have people around you whom you can rely on to host you for a few days if needed?	−9.47	0
11	In case of difficulties, do you have people around you whom you can rely on for material assistance?	−7.10	0
	Constant	75,14	

We collected patients' medical histories, clinical characteristics, treatments, and laboratory results (averaged over the three months preceding the study) from their medical records. No additional biological sampling or supplementary examinations were necessary for our study. All included patients were informed about the study's purpose and agreed to complete the EPICES questionnaire. The study was approved by the local Ethics Committee.

To differentiate elements dependent on healthcare providers from those dependent on patients, we evaluated Kt/V and hemoglobin (Hb) levels for the providers and the percentage of missed dialysis sessions for the patients. We considered that other factors were not strictly and exclusively dependent on one or the other.

In the absence of a defined deprivation threshold in this population of dialysis patients, we compared two groups of patients defined by the median EPICES score.

### Statistical methods

This study employed a cross-sectional design to evaluate the impact of individual socioeconomic deprivation on clinical care parameters and patient behaviors among maintenance hemodialysis patients. Statistical analyses were conducted using Stata 14 and a *p*-value < .05 was considered statistically significant.

#### Descriptive statistics

Patient characteristics were summarized using means and standard deviations (SD) for normally distributed continuous variables, and medians with interquartile ranges (IQR) for non-normally distributed variables. Categorical variables were expressed as frequencies and percentages.

#### Group stratification

Participants were stratified into two groups based on the median EPICES score (33.1):

Deprived (P+): EPICES score > 33.1
Non-deprived (P−): EPICES score ≤ 33.1

#### Group comparisons

Between-group differences (P + vs. P−) were assessed as follows:

- Continuous variables: Independent t-tests were used when assumptions of normality (Shapiro–Wilk test) and equal variances were met; otherwise, the Mann–Whitney U test was applied.
- Categorical variables: Chi-square tests or Fisher's exact tests were used, depending on expected frequencies.

#### Multivariable analysis

To explore independent associations between deprivation and outcome variables (e.g. smoking status, session adherence), logistic regression models were constructed. For continuous outcomes such as hemoglobin or nPCR, linear regression was used. All models were adjusted for potential confounders: age, sex, presence of diabetes, dialysis duration, and center effect (random intercepts).

#### Sensitivity and subgroup analyses

A sensitivity analysis using the standard EPICES cutoff (≥30) was performed. Additionally, stratified analyses by sex and age tertiles were conducted to explore interaction effects between deprivation and demographic variables.

All regression models reported odds ratios (OR) or mean differences with 95% confidence intervals (CI).

All analyses were conducted using Stata 15 (Stata corp, USA), with two-sided *p*-values < .05 considered statistically significant.

## RESULTS

**Figure 1:**
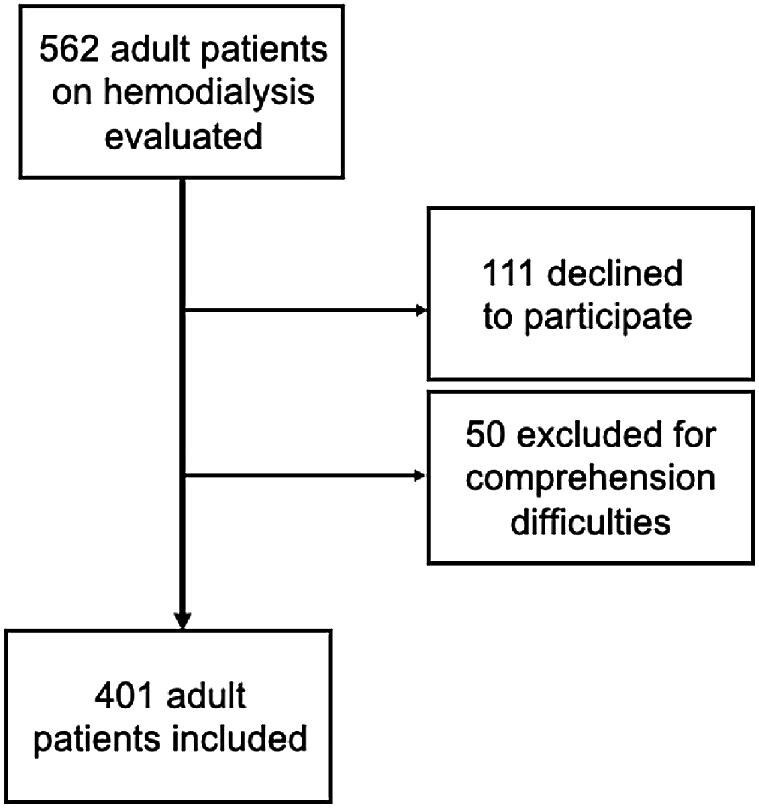
Flowchart of the study cohort.

Of the 562 adult patients approached for participation, 111 declined, and 50 were excluded due to comprehension difficulties or insufficient language proficiency. A total of 401 completed questionnaires were analyzed (Fig. 1).

The average age of participants was 68.5 years. The cohort comprised 60% men, 40.5% individuals with diabetes, and 92% with hypertension. Detailed demographic and clinical characteristics are presented in Table 2.

**Table 2:** Baseline characteristics of the study population (N = 401).

	Total Population	Non-precarious Population (P-)	Precarious Population (P+)	*P*
Number	401	200	201	NS
Age, mean (SD)	68.4 ± 0.7	68.98 ± 0.9	68.13 ± 0.9	NS
Sex, male (%)	60.3	63.8	56.9	NS
Weigth (kg)	78.1 ± 1.2	79.6 ± 1.4	77.6 ± 1.3	NS
BMI (Kg/m^2^)	28.2 ± 0.4	28.32 ± 0.4	28.2 ± 0.4	NS
Smoking (%)	25	15	34	.004
Hypertension (%)	92	92.4	91.6	NS
Diabetes (%)	40.5	39.9	41.1	NS
CAD (%)	22.25	21.7	22.8	NS
PAD (%)	16.8	14.7	18.8	NS
Stroke (%)	12.7	13.1	12.4	NS
Transplant listing (%)	23.2	25.1	21.3	NS
AVF (%)	94.75	96.5	93	NS
HDF (%)	55.9	51.8	59.9	NS
Dialysis vintage, months	74.9 ± 5.8	73.9 ± 6.1	76.3 ± 4.9	.02
EPICES score, median [IQR]	34.3 [21.9–47.9]	20.7 [14.2–28.1]	47.9 [39.1–56.8]	<.001

Legends: BMI: Body Mass Index; CAD: Coronary Artery Disease; PAD: Peripheral Artery Disease; AVF: arterio-venous fistula; HDF: Hemodiafiltration.

**Figure 2:**
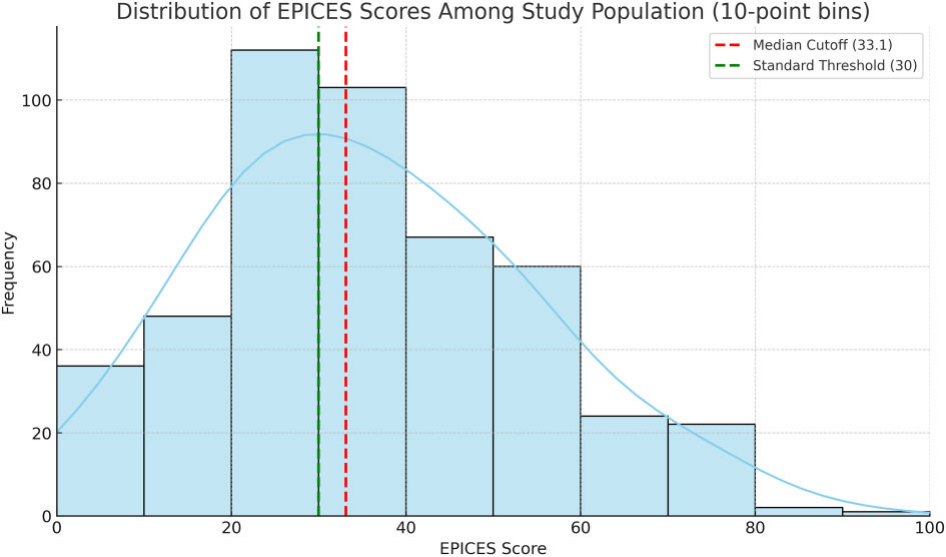
Histogram showing the distribution of EPICES scores among the study population. A kernel density curve is overlaid to illustrate the score dispersion. The red dashed line indicates the median cutoff (33.1), and the green dashed line shows the standard deprivation threshold (30).

The distribution of EPICES scores is shown in Fig. 2, exhibiting a right-skewed distribution with a concentration of patients below the standard deprivation threshold of 30. The median EPICES score was 33.1 [IQR: 21.9–47.9]. Based on this threshold, participants were stratified into two groups: non-deprived (P−, score ≤ 33.1) and deprived (P+, score > 33.1). The median score in the P − group was 20.7 [14.2–28.1] compared to 47.9 [39.1–56.8] in the P + group, a difference that was highly significant (Mann–Whitney U test, *p* < .0001).

Among the clinical and laboratory parameters assessed, the following were significantly different between groups:

- Dialysis duration was slightly longer in the P + group (76.3 vs. 73.9 months, *p* = .002),
- nPCR was higher in the P + group (1.28 vs. 1.06 g/kg/day, *p* = .007),
- Smoking was more frequent among P + patients (34% vs. 15%, *p* = .004),
- C-reactive protein (CRP) levels were also significantly elevated in the P + group (16.2 vs. 9.3 mg/L, *p* = .015).

No significant differences were observed in hemoglobin levels, erythropoietin (EPO) dosage, serum albumin, or rate of missed dialysis sessions (Table 3).

**Table 3:** Comparison of clinical and behavioral outcomes by deprivation status.

	Total Population	Non precarious Population (P-)	Precarious Population (P+)	*P*
Number	401	200	201	
Session duration (min)	243.2 ± 6	241.1 ± 3	245.6 ± 7.4	NS
Missed sessions	0.08 ± 0.03	0.06 ± 0.02	0.1 ± 0.05	NS
SBP (mmHg)	141 ± 1.2	142 ± 1.2	141 ± 1.4	NS
DBP (mmHg)	64 ± 1	63.3 ± 0.8	64.1 ± 0.9	NS
Number of anti-hypertensive drugs	0.98 ± 0.1	0.96 ± 0.1	0.99 ± 0.1	NS
ESA dosage (mcg/month)	95.4 ± 7.8	108.9 ± 9.9	89.4 ± 6.8	NS
Phosphate chelator dosage (mg/day)	2, 642 ± 198	2 809 ± 217	2 429 ± 196	NS
ONC (%)	18.9	16.1	21.8	NS
Serum albumin (g/L)	38.3 ± 4.3	38.3 ± 4.5	38.3 ± 4.2	NS
Kt/V	2.33 ± 0.65	2.36 ± 0.76	2.30 ± 0.76	NS
nPCR	1.14 ± 0.21	1.06 ± 0.05	1.28 ± 0.32	.007
K (mmol/l)	4.59 ± 0.02	4.61 ± 0.04	4.58 ± 0.04	NS
Ca (mmol/l)	2.35 ± 0.07	2.31 ± 0.1	2.22 ± 0.01	NS
PO3 (mmol/l)	1.45 ± 0.3	1.46 ± 0.3	1.44 ± 0.3	NS
Hemoglobin (g/dl)	11.5 ± 0.3	11.7 ± 0.5	11.2 ± 0.1	NS
Hematocrit (%)	35.2 ± 0.2	35.2 ± 0.25	35.1 ± 0.25	NS
CRP (mg/l)	11.3 ± 1.9	9.56 ± 0.8	14.3 ± 4.1	.02
HbA1c (%)	6.54 ± 0.15	6.53 ± 0.17	6.58 ± 0.14	NS

Legends: SBP: systolic blood pressure; DBP: Diastolic blood pressure; ESA: Erythropoietin Stimulating Agents; ONC: Oral Nutritional Complement.

In the multivariable analysis (Table 4), when modeling key outcomes as dependent variables and using socioeconomic deprivation (P + status) as the main independent predictor, we found that precarious patients were significantly more likely to be smokers (OR = 2.81; 95% CI: 1.55–5.12; *p* = .001), exhibited higher normalized protein catabolic rates (nPCR) (OR = 1.75; 95% CI: 1.12–2.95; *p* = .018), and had elevated CRP levels (OR = 1.04; 95% CI: 1.02–1.07; *p* = .003) after adjustment for age, sex, diabetes status, dialysis duration, and center effect. No significant differences were observed for serum albumin, hemoglobin concentration, or frequency of missed dialysis sessions. Additionally, a linear regression model testing the interaction between P + status and CRP on nPCR revealed a significant interaction term (OR = 1.06; 95% CI: 1.01–1.12; *p* = .025), suggesting that the association between socioeconomic deprivation and higher nPCR is amplified in the context of systemic inflammation (Table 4). This pattern persisted in a sensitivity analysis using the standard EPICES cutoff of 30 (Table 5).

**Table 4:** Multivariate regression models: association between precariousness (P+) as an independent variable and key outcomes (using median EPICES cutoff > 33.1).

Parameter	Odds Ratio (95% CI)	*P*-Value
Age (per 10 yrs)	0.92 (0.80–1.05)	.21
Male sex	0.84 (0.57–1.23)	.38
Diabetes	1.08 (0.74–1.59)	.69
Dialysis vintage (months)	1.01 (1.00–1.02)	.047
Smoking status (yes/no)	2.90 (1.60–5.30)	.001
CRP (mg/L)	1.04 (1.02–1.07)	.003
nPCR (g/kg/day)	1.75 (1.12–2.95)	.018
CRP × nPCR Interaction	1.06 (1.01–1.12)	.025

Deprived patients are ∼3x more likely to smoke. P + status associated with higher nPCR and higher CRP levels. The positive interaction term (P+) × CRP on nPCR suggests that the effect of precariousness on nPCR is amplified in patients with higher CRP, supporting the hypothesis of inflammation-driven hypercatabolism among deprived individuals.

All models adjusted for: age, sex, diabetes, dialysis vintage, and center effect.

**Table 5:** Sensitivity analysis using EPICES threshold ≥ 30.

Dependent Outcome	Adjusted Odds Ratio (95% CI)	*P*-Value
Smoking Status (Yes vs No)	2.72 (1.52–4.90)	.002
Elevated CRP (>10 mg/L)	2.34 (1.24–4.43)	.017
High nPCR (>1.2 g/kg/day)	1.98 (1.14–3.45)	.015
Interaction (CRP × nPCR High Levels)	1.86 (1.04–3.33)	.030

All models are adjusted for age, sex, diabetes status, dialysis vintage, and center effect. Precariousness (P+) defined by EPICES ≥ 30.

## DISCUSSION

This study offers new insights into the association between individual socioeconomic deprivation and the management of maintenance hemodialysis, using the EPICES index—a validated, individual-level measure of deprivation. In contrast to much of the existing literature, our findings suggest that clinical management, as delivered by healthcare teams, does not significantly differ between socioeconomically deprived (P+) and non-deprived (P−) patients in this cohort.

Across a comprehensive array of provider-driven indicators—including hemoglobin levels, erythropoietin and iron prescriptions, phosphate binder use, dialysis adequacy (Kt/V), and serum albumin—no statistically significant differences emerged between groups. These parameters are subject to close clinical monitoring and protocolized treatment within the French public healthcare system. Their uniformity suggests that individual socioeconomic status does not impact the delivery of evidence-based dialysis care in this setting.

Several patient-related variables, however, were notably different. Consistent with prior studies, the prevalence of tobacco use and elevated C-reactive protein (CRP) levels were significantly higher among deprived individuals, reinforcing the well-established links between social disadvantage, health-related behaviors, and chronic low-grade inflammation [18]. These findings support the hypothesis that behavioral and biological consequences of deprivation—rather than deficiencies in care—may contribute to differential outcomes in this population.

A particularly noteworthy finding was the significantly higher normalized protein catabolic rate (nPCR) observed among P + patients. Traditionally, nPCR is used as a surrogate for dietary protein intake, with higher values interpreted as reflecting better nutritional status. However, this interpretation may be misleading, particularly in the context of chronic inflammation. On Fresenius hemodialysis systems, nPCR is automatically estimated using integrated Online Clearance Monitoring (OCM) technology, which derives urea kinetics from intradialytic conductivity changes. While practical and widely used, this method does not differentiate between urea production from dietary intake versus endogenous protein breakdown. Inflammatory states, such as those prevalent among socially deprived patients, can induce hypercatabolism and increase protein turnover, leading to elevated urea generation and thus higher nPCR values—without corresponding improvements in nutritional intake [19].

In our cohort, this phenomenon is further supported by the concurrent elevation in CRP levels and smoking prevalence among P + individuals, suggesting a systemic pro-inflammatory milieu. The higher nPCR may therefore reflect inflammation-driven hypercatabolism rather than superior protein intake [20]. This highlights a limitation of nPCR as a nutritional marker, particularly in populations with high inflammatory burden, and underscores the need to interpret this parameter in the context of inflammatory biomarkers and clinical status. Although the prescription of oral nutritional supplements was more frequent in the P + group, this difference did not reach statistical significance and may not fully account for the observed nPCR differences.

To further validate and refine these interpretations, we used regression models to treat precariousness as the independent variable, more accurately reflecting the hypothesized causal relationship. In addition, we formally tested for an interaction between socioeconomic status and inflammation on protein metabolism, and conducted a full sensitivity analysis using the validated EPICES ≥ 30 threshold. These updates not only enhance the interpretability of our findings but also reinforce the methodological rigor and generalizability of our study.

In contrast, serum albumin concentrations did not differ significantly between groups. While often used as a nutritional indicator, albumin is influenced by multiple non-nutritional factors, including inflammation, liver function, and hydration status. Its relative insensitivity in this context further emphasizes the limitations of single-parameter nutritional assessments and calls for a multidimensional approach incorporating both intake and catabolic markers.

Interestingly, despite trends suggesting a slightly higher proportion of missed dialysis sessions in the P + group, this difference did not reach statistical significance. This may reflect successful mitigation strategies within our healthcare system, including social support and transportation assistance, which help to ensure treatment adherence even among vulnerable populations.

The use of the EPICES score represents a methodological strength of this study. Unlike area-based deprivation indices, which may obscure within-neighborhood variability, the EPICES instrument captures individual-level dimensions of social vulnerability—including financial hardship, social isolation, and limited access to cultural or recreational activities. This granularity likely enhances the sensitivity of our analyses and contributes to our divergence from studies conducted in other healthcare settings, particularly those relying on geographically aggregated socioeconomic data.

It is also plausible that differences in healthcare systems contribute to these divergent findings. For example, the only other study that failed to identify a negative impact of deprivation on dialysis outcomes was also conducted in France [4]. In contrast, studies in countries with more fragmented or market-based health systems—such as the United States—often show stronger associations between deprivation and mortality or under-treatment. Interestingly, in the U.S., the link between deprivation and mortality diminishes after age 65, when access to universal Medicare coverage begins [6].

Our findings reinforce the relevance of individual-level deprivation in explaining certain patient behaviors, such as smoking and potentially dietary intake, while also illustrating the equity of care delivery across socioeconomic strata in a system with strong public healthcare infrastructure.

Nonetheless, certain limitations warrant consideration. This study was conducted in an economically privileged region—one of the wealthiest in France. As such, our findings may not be generalizable to more disadvantaged areas, where structural barriers may play a more prominent role. Another limitation of our study is the exclusion of patients with comprehension difficulties or insufficient language proficiency, who may also represent some of the most socioeconomically vulnerable individuals. This inherent limitation of self-administered tools such as the EPICES questionnaire may have led to an underestimation of the true impact of deprivation within the hemodialysis population.

Despite these limitations, this study stands out for its focus on a highly relevant and underexplored dimension of health equity: the role of individual socioeconomic status in shaping dialysis care. By moving beyond geographic proxies and employing a direct measure of deprivation, we offer a more nuanced understanding of how social factors interact with biological, behavioral, and medical dimensions of care.

## CONCLUSION

This study is the first to evaluate the impact of socioeconomic deprivation on the management of hemodialysis patients using an individual-level assessment through the EPICES index. Our findings reveal no major differences in clinical management between deprived and non-deprived patients, suggesting equitable care delivery within the studied population.

These results diverge from previously published data, which often report disparities linked to socioeconomic status. This discrepancy may stem from methodological differences, particularly our use of individualized deprivation metrics, as well as systemic differences in healthcare and social protection across countries. The relatively uniform management observed in our cohort may reflect the strengths of the French healthcare system in promoting equal access to care.

Further studies across diverse settings are warranted to confirm these findings and to explore the nuanced ways in which deprivation influences patient behavior, biological markers, and long-term outcomes in end-stage renal disease.

## Data Availability

The data underlying this article are not publicly available but will be shared on reasonable request to Dr Yves Dimitrov.
